# Proline 411 biases the conformation of the intrinsically disordered plant UVR8 photoreceptor C27 domain altering the functional properties of the peptide

**DOI:** 10.1038/s41598-018-37005-8

**Published:** 2019-01-28

**Authors:** Min Wu, Daniel Farkas, Leif A. Eriksson, Åke Strid

**Affiliations:** 1 Department of Chemistry and Molecular Biology, University of Gothenburg, SE-40530 Gothenburg, Sweden; 2 School of Science and Technology, Örebro University, SE-70182 Örebro, Sweden; 3 Present Address: Department of Biology and Biological engineering, Chalmers University of Technology, SE-41296 Gothenburg, Sweden

## Abstract

UVR8 (UV RESISTANCE LOCUS 8) is a UV-B photoreceptor responsible for initiating UV-B signalling in plants. UVR8 is a homodimer in its signalling inactive form. Upon absorption of UV radiation, the protein monomerizes into its photoactivated state. In the monomeric form, UVR8 binds the E3 ubiquitin ligase COP1 (CONSTITUTIVELY PHOTOMORPHOGENIC 1), triggering subsequent UV-B-dependent photomorphogenic development in plants. Recent *in vivo* experiments have shown that the UVR8 C-terminal region (aa 397–423; UVR8^C27^) alone is sufficient to regulate the activity of COP1. In this work, CD spectroscopy and NMR experiments showed that the UVR8^C27^ domain was non-structured but gained secondary structure at higher temperatures leading to increased order. Bias-exchange metadynamics simulations were also performed to evaluate the free energy landscape of UVR8^C27^. An inverted free energy landscape was revealed, with a disordered structure in the global energy minimum. Flanking the global energy minimum, more structured states were found at higher energies. Furthermore, stabilization of the low energy disordered state was attributed to a proline residue, P411, as evident from P411A mutant data. P411 is also a key residue in UVR8 binding to COP1. UVR8^C27^ is therefore structurally competent to function as a molecular switch for interaction of UVR8 with different binding partners since at higher free energies different structural conformations are being induced in this peptide. P411 has a key role for this function.

## Introduction

Although UV-B (280–315 nm) is only a small fraction of the total solar radiation at the earth’s surface, it has a significant impact on life on our planet. In plants, UV-B serves as an inducer, evoking critical physiological responses ranging from photomorphogenesis and photoprotection to circadian rhythms. This includes both molecular and intracellular changes^1–4^, such as induction of expression of at least 100 different genes, the accumulation of flavonoid-derived pigments^5^ and antioxidants, DNA repair, as well as alteration of plant morphology^6^.

Recently, the UV RESISTANCE LOCUS 8 (UVR8) was identified as a unique UV-B photoreceptor^7^. UVR8 exists as a homodimer in the absence of UV-B. It is only upon the absorption of UV-B radiation that UVR8 monomerizes and is activated as a signalling molecule^7^ and tryptophan residues of the Trp-rich UVR8 polypeptide have been shown to be chromophores for the UV-B absorption^7,8^. It has been shown that closely stacked tryptophan residues W285, W233, and W337, located at the interface of the UVR8 dimer, are responsible for the perception of UV-B radiation^9,10^. At the interface of the homodimer complex, charged residues are also found arranged in patches of complementary electrostatic potential so as to form a network of salt bridges that hold the two UVR8 units together^9,10^. Most likely, a proton-coupled electron-transfer reaction triggered by the UV-B absorption results in the neutralization and breakage of some essential interfacial salt-bridges, leading to the dissociation of the UVR8 dimer complex^11,12^. In the UVR8 three-dimensional structure obtained by X-ray crystallography^9,10^, the C-terminal domain is missing. In fact, this peptide had to be removed before the UVR8 protein lent itself to crystallization. Instead, the structure was dominated by a central core consisting of two seven-bladed propeller RCC1 folds each contributed from a separate UVR8 monomer. Notwithstanding, the dimer of truncated UVR8 was still responsive to UV-B and was monomerized in the same way as the full-length protein^13^. Thus, the C-terminal peptide was not involved in the initial signalling events consisting of UV absorption and breaking of the interactions holding the UVR8 dimer together.

Furthermore, it has been found that UV-B stimulates nuclear accumulation of the monomeric UVR8, resulting in the expression of the *HY5* gene which encodes the crucial HY5 transcriptional regulator of the UVR8 pathway^14,15^. Recently, the E3 ubiquitin ligase CONSTITUTIVELY PHOTOMORPHOGENIC 1 (COP1), a protein that contains a WD40-repeat domain^16^, was identified to be essential for UVR8 nuclear import^14^. After absorption of UV quanta and monomerization of UVR8, UV-B signalling is further transmitted by the activated UVR8 monomer through its direct interaction with COP1^3^. This is accomplished through two of its domains, a part of the C-terminal domain encompassing residues 397–423 (hereafter denoted UVR8^C27^) and the central RCC1 domain where the UVR8^C27^ domain previously has been shown to be essential for the interaction with COP1^13,17^. In fact, in a recent study by Yin *et al*.^17^, the UVR8^C27^ domain was shown to be sufficient on its own in mimicking the action of the full size UVR8 monomer with regards to its action on COP1, i.e. being able to modulate the activity of COP1 and downstream signalling *in vivo*, including *HY5* expression. Thus, from a signalling point of view, the bulk of the UVR8 protein is not needed to accomplish further activity once it has formed the heterodimer together with COP1 and been transported into the nucleus. Instead, the UVR8^C27^ peptide is now the active entity. In plants, the COP1 protein acts as a switching hub orchestrating several different light-induced developmental processes^18^. However, in humans, where a COP1 orthologue also exists, it is involved in tumorigenesis. In the processes in *Homo sapiens*, interactions of COP1 with several other proteins are linked to a specific VP amino acid residue motif of the interaction partners^16^. Previously, we have in fact also in plants shown^19^ that UVR8^C27^, in addition to other COP1-binding proteins^20,21^, contains a conserved VP motif (V410 and P411). These two hydrophobic residues were later shown to be essential for the COP1 binding to occur^13^. In summary, the functional aspects of COP1, such as its activities, and its interactions with different upstream and downstream components therefore is of broad scientific interest, from plant biology to medicine.

In the dark, UV-B signalling is suppressed by REPRESSORS OF UV-B PHOTOMORPHOGENESIS 1 and 2 (RUP1 and RUP2). These proteins facilitate the re-dimerization of the UVR8 monomer. UVR8 and RUPs directly interact through the UVR8^C27^ domain. This action rapidly inactivates the regulatory function of UVR8 on plant gene expression^22,23^. The interplay between the RUPs and COP1, and the mechanism by which UVR8^C27^ discriminate between the two different interaction partners, is still unknown. Very little structural information is available for the UVR8^C27^ domain, albeit X-ray crystal structures have been published^9,10^ for UVR8 in its inactivated homodimer form, where, as discussed above, the C-terminal region including the COP1-binding domain (UVR8^C27^) is missing. In our previous theoretical work, we predicted that the UVR8^C27^ domain is rather flexible and that the two residues V410 and P411 play a key role in the formation of the heterodimeric complex with COP1^19^.

In the work presented here, the structural dynamics of the UVR8^C27^ domain is investigated using both atomic meta-dynamics simulations and different spectroscopic techniques with the aim to understand the function of the UVR8^C27^ domain and the structural significance of the VP-motif. The fact that the UVR8^C27^ domain and the rest of the UVR8 protein are functionally distinct (i.e. the UVR8^C27^ domain is not needed for the initial UV absorption and monomerization processes and UVR8^C27^ is on its own sufficient for inducing downstream signalling through COP1; see above), we used the isolated UVR8^C27^ peptide for these studies, which considerably simplifies procedures. We find that UVR8^C27^ is intrinsically disordered and has the ability to adopt a large number of different random secondary structures in its ground state. The peptide gains more structure with increasing energy. Such a high energy state would *in situ* likely be obtained in an encounter complex between UVR8^C27^ and a larger protein to which it has affinity (e.g. COP1 or RUP). Crucial for the intrinsic disorder is amino acid P411 that we demonstrate has the ability to break the formation of an extended α-helix in the UVR8^C27^ peptide, which can appear when P411 is exchanged with an alanine.

## Results

### Structural characterization of the UVR8^C27^ domain

UVR8 is in its inactivated homodimer form not able to bind COP1. Upon absorption of UV radiation, the UVR8 homodimer undergoes conformational rearrangements that ultimately lead to dimer disruption and formation of activated UVR8 monomers. In its monomeric state, UVR8 is able to initiate UV-signalling through the interaction of the UVR8^C27^ domain with COP1. Upon return to dark conditions, COP1 is replaced by a RUP monomer and the inactivated UVR8 homodimer is later re-established. These sequential monomerization/dimerization events can most likely be explained by the conformation/availability of the UVR8 C-terminus for binding to the different interaction partners (COP1, RUP1/2, or a second UVR8), and how the UVR8^C27^ domain is structurally rearranged during these different states. In this paper, a structural and dynamic characterization of this domain was performed to ascertain the functional role of UVR8^C27^, including the implications this has for modulation of the interactions with COP1 and the RUPs.

As a first step in the pursuit of understanding this complex sequence of events, we focused our attention to the UVR8^C27^ peptide and characterized this domain by far-UV circular dichroism (CD) spectroscopy. Since protein interactions are strongly coupled to the structure of the interacting partners, it was highly desirable to establish the structural behaviour of the UVR8^C27^ domain. The capability of UVR8^C27^ to specifically interact with several different interactions partners suggests the presence of different conformations of the peptide. Thus, by probing its structural changes using far-UV CD with increasing temperatures, clues to such conformations could potentially be obtained. Increases in temperature is one way of increasing the free energy of the peptide. This high energy state may mimic the structural precursor of UVR8 ^C27^ that is involved in establishing the “encounter complex” formed between the peptide and a possible interactive protein (e.g. COP1).

**Figure 1:**
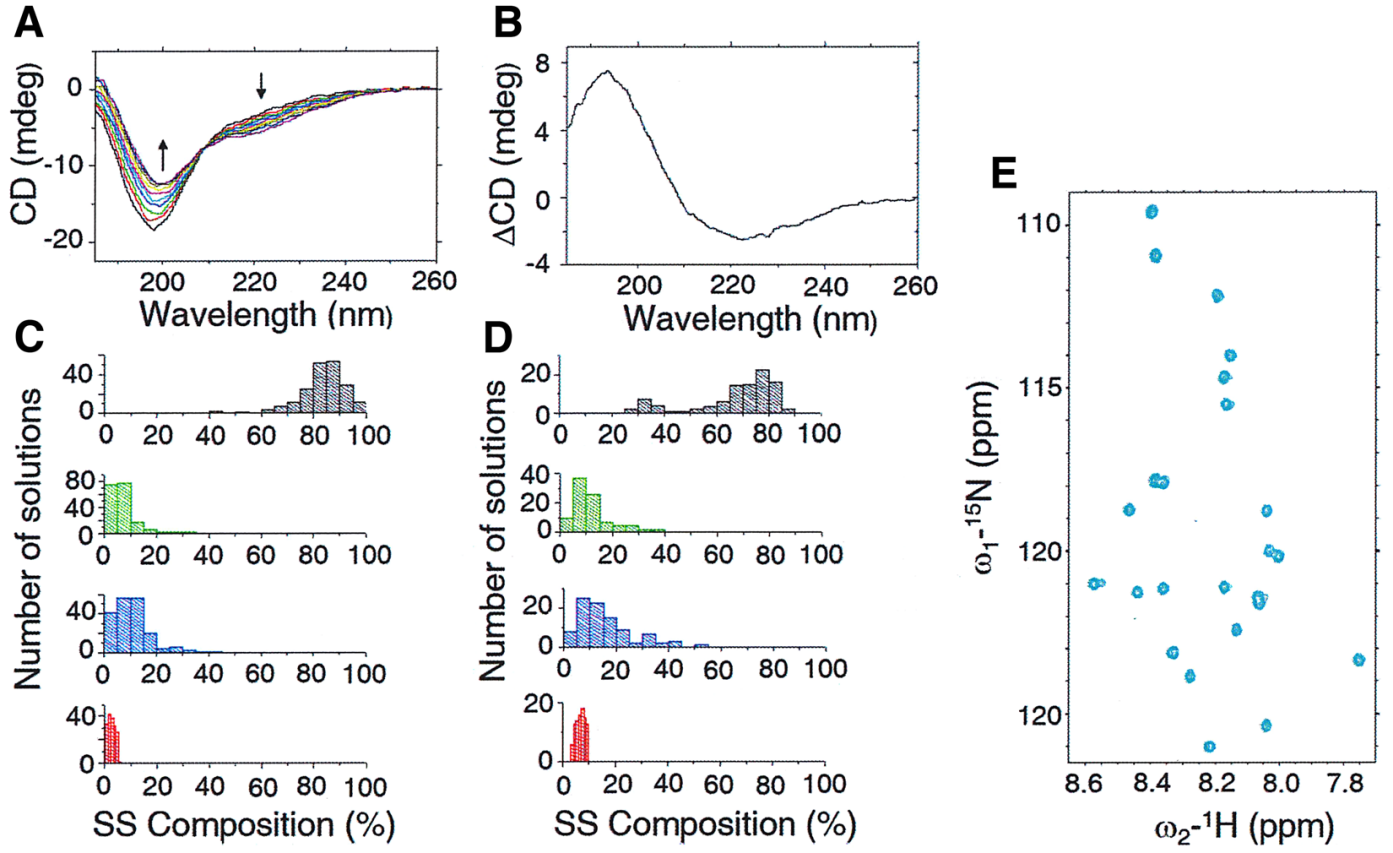
Structural characterizations of the UVR8^C27^ peptide. (**A**) Far UV-CD spectra recorded at varying temperatures for the UVR8^C27^ peptide. The direction of the spectral changes with increasing temperature are indicated with arrows. All spectra were recorded for a 33 µM solution in deionized water at pH6. (**B**) Difference between far UV-CD spectra recorded at 98 °C and 10 °C, respectively, for UVR8^C27^. (**C**,**D**) Secondary structure (SS) compositions calculated from reconstructed CD spectra for the UVR8^C27^ peptide at 10 °C (**C**) and 98 °C (**D**), respectively. The distribution of all valid solutions is shown. Disordered structures are shown in grey, turns in green, β-strands in blue and α-helices in red. (**E**) Naturally abundant ^1^H -^15^N HSQC NMR spectrum of the UVR8^C27^ peptide recorded for a 2 mM sample at 25 °C in 10% D_2_O at pH6. Side-chain amide groups have been omitted in this view.

Our measurements showed characteristic intrinsically disordered protein (IDP)-like features for the UVR8^C27^ peptide, with a strong negative peak at 198 nm and a negative shoulder around 220 nm (Fig. 1A). A gradually more shallow negative peak at 198 nm and a more pronounced negative shoulder around 220 nm was found at increasing temperatures (10–98 °C), Fig. 1A. Furthermore, a slight but distinct shift towards longer wavelengths for the negative peak around 198 nm occurred. As indicated by the difference spectrum (Fig. 1B), spectral features consisting of a strong positive peak around 193 nm and a broad negative dip around 223 nm were gradually obtained with increasing temperature. Interestingly, the difference spectrum is consistent with a gain in β-sheet structure as the temperature increased. However, it is not possible to rule out the presence of other structural elements due to regional overlap. It is worth noting that the characteristic IDP spectrum recorded at 10 °C could be fully recovered after thermal annealing, thus excluding any tendency of aggregation of the UVR8^C27^ domain at high temperatures.

A single isodichroic point at 209 nm could be observed for UVR8^C27^ during the temperature scan. Reconstructions of data sets recorded at 10 °C and 98 °C (Fig. 1C,D) show the distribution of secondary structure elements of the UVR8^C27^ peptide. The average measured secondary structures are 3% α-helix, 9% β-strand, 6% turns and 82% disordered structure at 10 °C, and 6% α-helix, 15% β-strand, 11% turns, and 67% disordered structure at 98 °C **(**SI Appendix, Table S1). The largest structural changes were observed in the content of β-structure, increasing from 9 to 15%. This is also evident from the difference spectrum (Fig. 1B). The second largest change is observed in turns increasing from 6 to 11%. The α-helix content doubled from 3 to 6%. Our data imply that, as the temperature is raised (i.e. as the free energy of the peptide increases), the disordered peptide transiently access conformations containing ordered secondary structure.

The naturally abundant ^1^H-^15^N HSQC NMR spectrum for UVR8^C27^ recorded at 25  °C in 10% D_2_O at pH6 is shown in Fig. 1E. From this spectrum it is obvious that all 24 backbone NH-groups (not counting prolines and the N-terminal residue) can be found within the same narrow spectral window (8.6 ppm-7.7 ppm) in the ^1^H-dimension. The indicated spectral range is typical for intrinsically disordered proteins reflecting the dynamic nature of the peptide^24,25^.

### Bias-exchange metadynamics simulations of the UVR8^C27^ domain

Examination of the UVR8^C27^ CD spectra as a function of temperature (Fig. 1) strongly indicates that at higher temperatures, i.e. at higher free energies, the peptide can adopt conformations with more ordered secondary structure. In order to study this interesting behaviour, bias-exchange metadynamics simulations were performed.

#### Free energy landscape of UVR8^C27^

**Figure 2:**
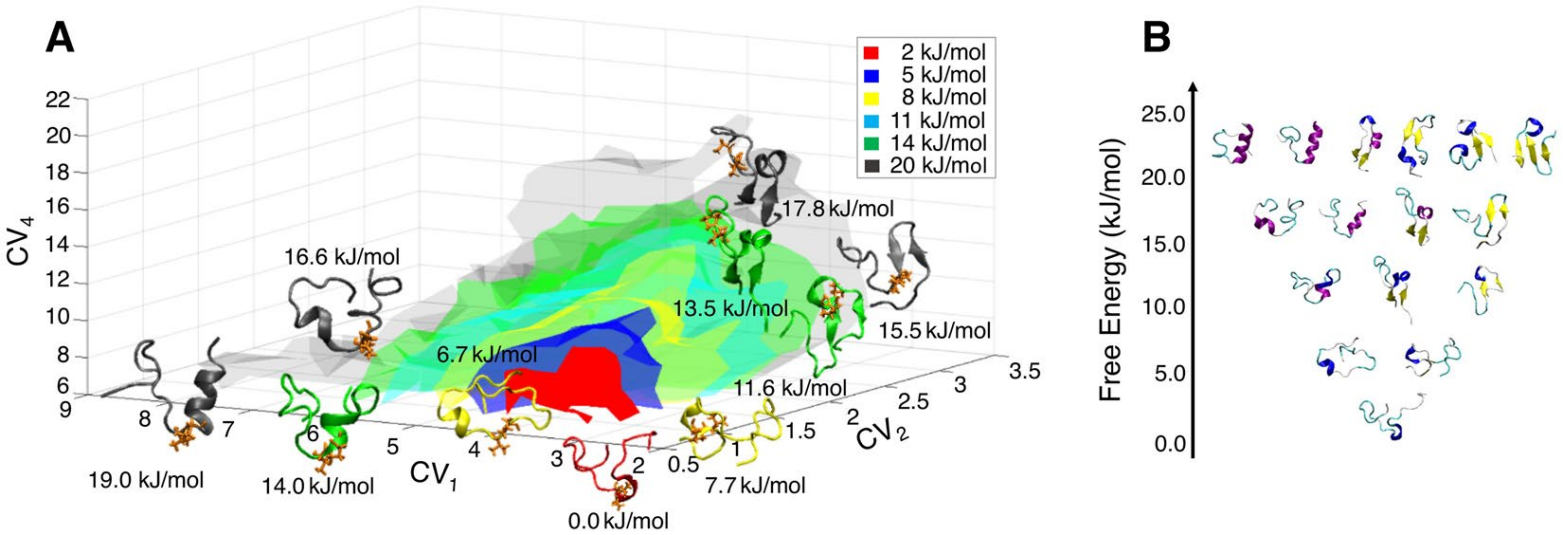
(**A**) Three-dimensional representation of the free energy landscape of the UVR8^C27^ peptide as a function of three collective variables (CV_1_, CV_2_ and CV_4_; see the Methodologies section for definitions of the collective variables). Isosurfaces are shown at 2 (red), 5 (blue), 8 (yellow), 11 (cyan), 14 (green) and 20 kJ/mol (grey). Representative structures sampled during the simulation are also shown along with their relative free energies. (**B**) Characterization of the free energy structures of the UVR8^C27^ peptide. Representative conformations sampled during the simulations are shown at increasing free energies with respect to the structurally disordered global minimum. The energy of the global minimum is set to zero, and all other structures computed relative to this.

A wide range of conformations were produced in the bias-exchange metadynamics simulations. In Fig. 2, the free energy landscape of the UVR8^C27^ domain is presented as a function of the three variables antiparallel β-sheet content, α-helix content, and number of hydrophobic contacts. The global free energy minimum (red area) is degenerate in this landscape, corresponding to an ensemble of intrinsically disordered structures with only a few transient tertiary contacts and almost no secondary structure elements. A similar free energy landscape was obtained using the parallel β-sheet content instead of the antiparallel β-sheet content as one of the collective variables, see SI Appendix, Fig. S3. As the free energy increased, a wide variety of partially structured conformations emerged. Their free energies are in the range of 5–10 kJ/mol higher than that of the global minimum which indicates that their existence cannot be neglected. These structures are characterized by different secondary structures including α-helices, β-sheets, and semi-disordered polyproline helices II (PPII) in different combinations, cf. Fig. 2B. There is no sizeable free energy barrier for the formation of these states and thus they are all kinetically connected to the disordered free energy minimum and exchange can occur rapidly between them. This complex behaviour agrees with the single isodichroic point observed for C27 in CD measurements.

**Figure 3:**
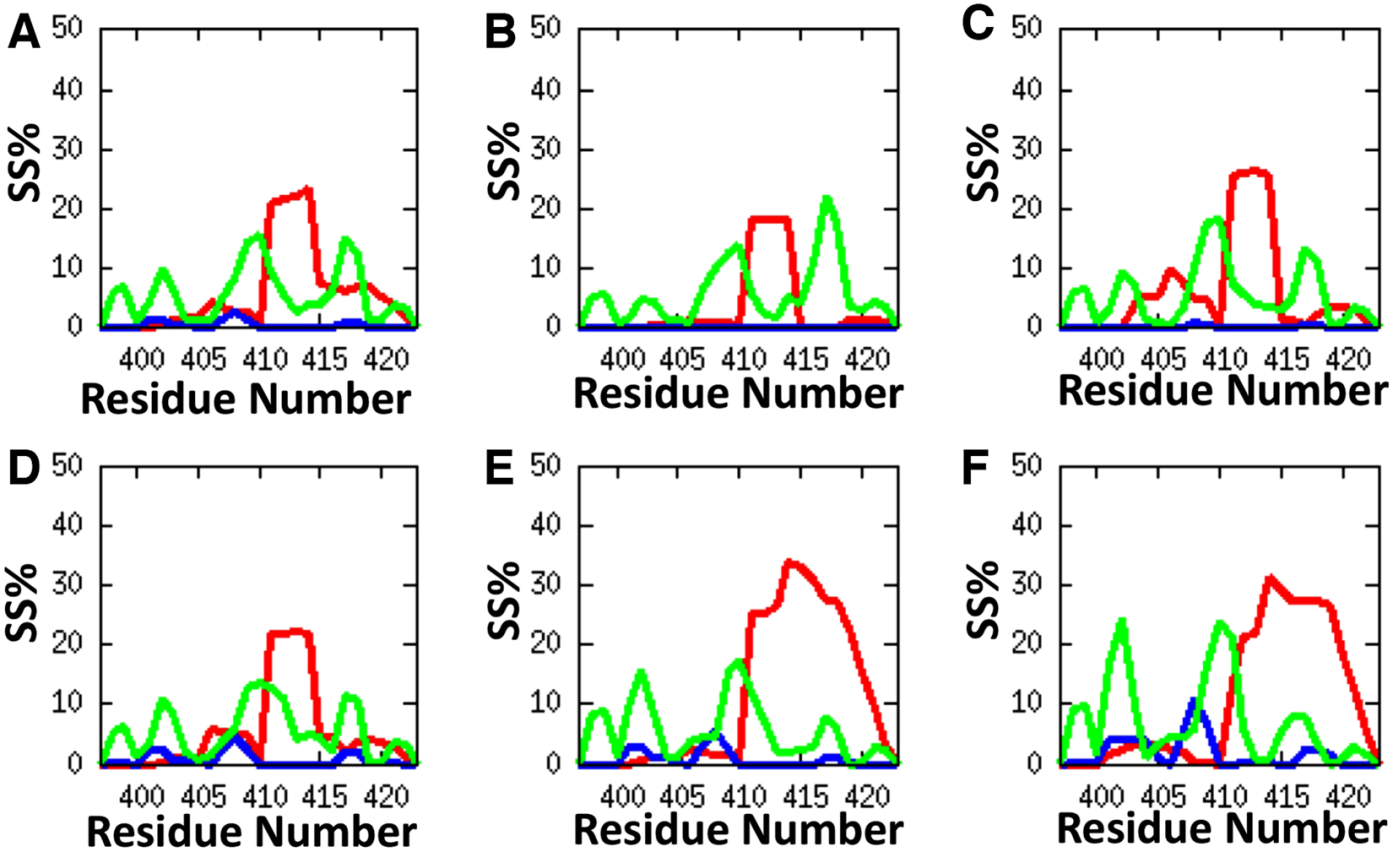
Residue-based analysis of the different secondary structure (SS) populations for the UVR8^C27^ domain at increasing relative free energies. α-Helical regions are shown in red, β-sheet regions in blue and polyproline II structures (PPII) in green. The y-axis shows the fraction of the total population that is present in this particular free energy interval. (**A**) the region 0–25 kJ/mol showing the average for all windows (**B–F**) where (**B**) corresponds to 0–5 kJ/mol, (**C**) to 5–10 kJ/mol, (**D**) to 10–15 kJ/mol, (**E**) to15–20 kJ/mol, and (**F**) to 20–25 kJ/mol.

The fractions of the total population present as α and β secondary structures were calculated for each amino acid residue of the UVR8^C27^ domain using the DSSP programme^26^ for different free energy windows (Fig. 3). The composition of PPII structure is also included, since it is frequently observed in IDPs and found to be favourable for protein-protein and protein-nucleic acid interactions. IDPs also play major roles in signal transduction and protein complex assembly^27,28^. The PPII structure is defined in the coil region for at least two consecutive amino acids with the dihedral angles φ and ψ in the range (φ ± ε, ψ ± ε), with φ, ψ and *ε* being −75°, 145°, and 29°, respectively^29^. α-helical conformations are preferred compared to β-strand conformations in the lower free energy regions, albeit with an overall low secondary structure content. This is consistent with our previous theoretical results^19^. The average population of α-helical conformations is approximately 3.2%, whereas it is 0.0% and 5.7% for β and PPII structures, respectively, in the lowest free energy regime (0–5 kJ/mol; Fig. 3B). As the free energy progressively increases, the amount of α-helical, anti-parallel β-sheet and PPII secondary structure elements increase accordingly, following a characteristic sequence-dependent pattern. At the free energy up to 5 kJ/mol above the minimum, residues P411-T414 have reached a probability of about 20% to form an α-helical secondary structure conformation. For the free energy range 5–10 kJ/mol above the minimum, the α-helical content increases and can be found also in the regions A404-V409 and D418-K422. As the free energy increases above 15 kJ/mol (Fig. 3E,F), the α−helical conformations are mainly formed in the second half of the peptide. In our previous work, the conformational predictions for UVR8^C27^ were evaluated by a static scoring function and the α-helical conformations were predicted to be localized mainly at A404-V409 which in this study, however, is obtained at a relatively high free energy range (5–10 kJ/mol; Fig. 3C). β-sheet structures start to appear only at free energies above 10 kJ/mol (Fig. 3D–F) and are then mainly located in the first half of the peptide, as was also found in our previous work^19^. For the 0–15 kJ/mol free energy windows (Fig. 3B–D), PPII structures can be found at several different locations in the peptide. Based on the percentages, major and minor regions of PPII structure can be observed. Two major regions can be identified at the segments Y407-P411 and G415-D418, and three minor regions at K398-S399, S402-P403, and S420-K422.

#### Solvent exposed surface and hydrodynamic properties of the UVR8^C27^ domain

**Figure 4:**
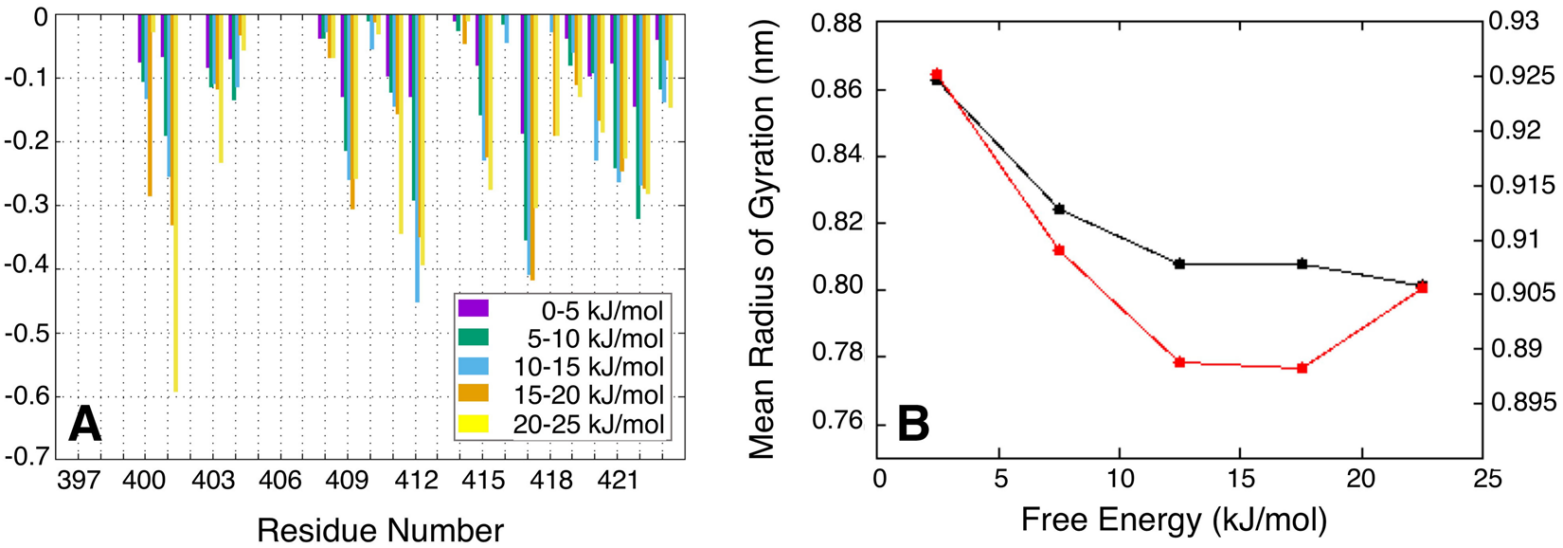
The UVR8^C27^ domain becomes more compact at higher energies. (**A**) Difference in solvent accessible surface area (ΔSASAs) for selected residues in the UVR8^C27^ domain, colour coded with reference to the different free energy windows according to the list in the lower right-hand corner. The bars correspond to the mean SASA difference obtained by subtracting the SASA average at the global free energy minimum (0 kJ/mol) from the values in the different free energy windows. (**B**) Mean radius of gyration (black line) and SASA value (red line) of the UVR8^C27^ peptide as functions of the free energy.

Differences in residue-based solvent accessible surface area (ΔSASA) for the UVR8^C27^ domain were calculated for the different free energy windows by subtracting the SASA average obtained at the global free energy minimum (0 kJ/mol). The results are shown in Fig. 4A. The average SASA and the radius of gyration of the UVR8^C27^ peptide as functions of the free energy are shown in Fig. 4B. There is a dramatic decrease in the average SASA and the radius of gyration for UVR8^C27^ as the free energy increases. This suggests that the UVR8^C27^ domain becomes more compact at higher energies, which is consistent with the observed increase in secondary structure. The significant SASA reductions are not restricted to hydrophobic residues (W400, V401, P403, A404, V409, P411, G415, G419 and G423) but are also seen for charged (D412 and K422) and polar (T417, S420 and S421) amino acids.

### A comparison between theoretical simulations and experimental results

The architecture of the free energy landscape described above illustrates an intrinsically disordered structure at the global free energy minimum. Consistent with CD experiments, simulations show that as the free energy increase, the UVR8^C27^ peptide becomes transiently more structured. Furthermore, the detailed information obtained from the meta-dynamics simulations highlight the VP-motif (consistent with our previous findings; ref.^19^) and how this biases the system to form a PPII structure, which suggests conserved structural elements for efficient binding to COP1 and/or the RUPs, cf. Fig. 3. Proline has previously been found in many other intrinsically disordered structures^30–32^. We suggest that P411 of the VP-motif stabilizes the PPII structure of the UVR8^C27^ peptide, thus maintaining the disordered structure of UVR8^C27^ peptide.

### Structural significance of the VP motif

The single proline residue, P411, was indicated to have a significant role in the stabilization of the non-structured/PPII region (aa 397–411), as evident from Fig. 3. The P411 residue appears to block the propagation of an α-helix structural element from the C-terminal side into the first half of the UVR8^C27^ peptide. We thus decided to characterize the mutant peptide UVR8^C27^ P411A (UVR8^C27P411A^) by CD spectroscopy. Interestingly, the UVR8^C27P411A^ peptide has very similar spectral features as the UVR8^C27^ peptide, showing a large negative peak at 198 nm and a negative shoulder stretching past 220 nm (SI Appendix, Fig. S4A). As temperatures and free energy increased, the secondary structure content of the UVR8^C27P411A^ peptide mutant also increased in a manner similar to the wild type UVR8^C27^ peptide. An isodichroic point was also noted at 208 nm for the thermally induced folding of the UVR8^C27P411A^ peptide. The difference spectrum of the UVR8^C27P411A^ peptide (SI Appendix, Fig. S4B) reveals similar spectral features as for the wild type UVR8^C27^ peptide, suggesting the formation of similar structural elements upon heating. Indeed, spectral reconstruction of UVR8^C27P411A^ peptide data shows an increasing secondary structure composition in similar proportions as for the UVR8^C27^ peptide (compare Fig. 1C,D with SI Appendix, Fig. S4C,D).

**Figure 5:**
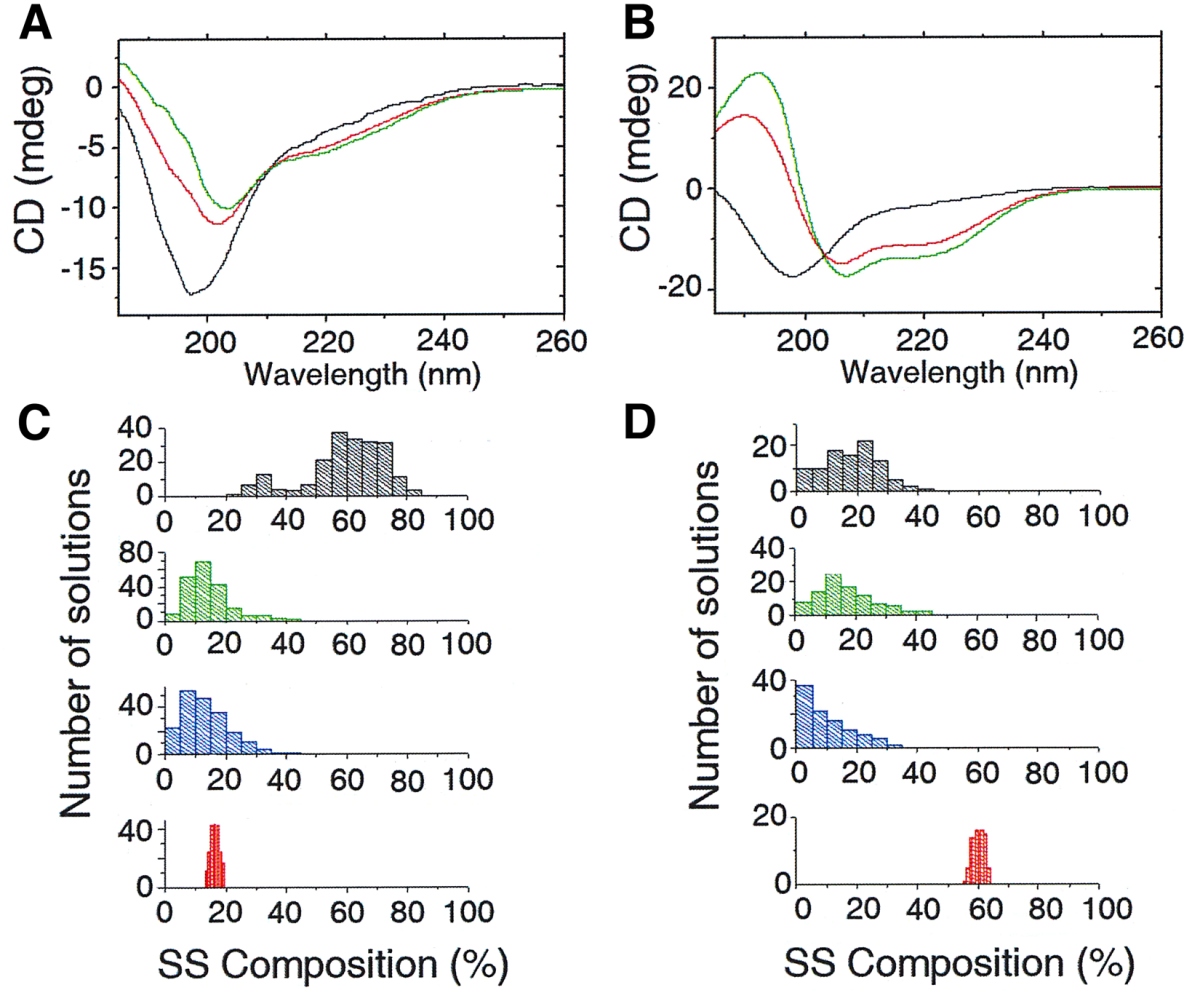
The effect of trifluoroethanol on the structure of UVR8^C27^ peptide. Far-UV CD spectra recorded for (**A**) UVR8^C27^ and (**B**) UVR8^C27P411A^ peptides in presence of 0% trifluoroethanol (TFE; black), 25% TFE (red), and 50% TFE (green) at 20 °C. The secondary structure (SS) composition was calculated for (**C**) UVR8^C27^ and (**D**) UVR8^C27P411A^ in the presence of 50% TFE. The distributions of all valid solutions are shown. Disordered structures are shown in grey, turns in green, β-strands in blue and α-helices in red.

However, there are other ways of probing the propensity to form secondary structure that does not necessarily involve elevated temperatures. Trifluoroethanol (TFE) is a co-solvent commonly used in folding studies and that is known to shield off molecular interactions between surrounding water molecules and the peptide backbone, thus assisting the peptide to form a more ordered structure by the formation of intramolecular hydrogen bonds. This compound is thus suitable for probing the readiness of the peptide to form secondary structure elements^33^. In Fig. 5A, the CD spectra for the UVR8^C27^peptide is shown at gradually increasing concentrations of TFE (0, 25, and 50%) at 20 °C. Indeed, TFE induced structural changes in UVR8^C27^, giving analogous but slightly exaggerated features for the TFE-spectra to what was seen in the high temperature spectrum shown in Fig. 1A. A similar, but not identical, isodichroic point is observed at 210 nm comparing the two figures. From spectral reconstruction, similar trends were also noted for UVR8^C27^ in TFE as compared with the high temperature spectrum, showing an evident redistribution of α-helical and β-sheet contents with a notably larger gain in α-helical structure (Fig. 5C; SI Appendix, Table S2), much in agreement with the theoretically predicted behaviour of UVR8^C27^ (see previous section).

As is evident from Fig. 5B, and in sharp contrast to the case for the UVR8^C27^ domain, the UVR8^C27P411A^ peptide exhibited a significant structural change in presence of TFE. The observed UVR8^C27P411A^/TFE spectra (25%-50% TFE) are typical for an α-helix-containing peptide, showing a large positive peak around 192 nm and two negative dips at 207 nm and 222 nm. From spectral reconstruction of the UVR8^C27P411A^ peptide’s CD spectrum, recorded in the presence of 25 (SI Appendix, Table S2) and 50% (Fig. 5D and SI Appendix, Table S2) TFE, a step-wise increase in α-helix content occurred, corresponding to increases up to 42 and 60% of the entire secondary structure of the UVR8^C27P411A^ peptide, respectively. This is in sharp contrast to only 8 and 16% α-helical content, respectively, for the wild type C27 peptide under the same conditions (Fig. 5C).

## Discussion

Experimental characterization of UVR8^C27^ by far-UV CD and NMR revealed IDP-like features of this domain. The UVR8^C27^ domain is structurally disordered at low temperature but becomes more structured as the temperature is elevated and thereby as its free energy increases, as is evident from CD difference spectra and data reconstructions. From the metadynamics simulations, the observed single isodichroic point in the thermal scan suggest the presence of an ensemble of transiently accessible conformations of C27 with more secondary structure at higher energy (temperature). The increase in secondary structure in UVR8^C27^ as its free energy increases is consistent with a gain in structure as the peptide forms an encounter complex with a larger interaction partner before stable binding.

In the theoretical studies, bias-exchange metadynamics simulations were performed. An inverted free energy landscape of UVR8^C27^ was observed after the simulation had converged. Similar characterizations have also been carried out for other intrinsically disordered proteins, such as the Aβ40 peptide^34^. We found a largely disordered structure at the global minimum and a variety of partially folded conformations appearing at 5–10 kJ/mol of free energy above the minimum, supporting the experimental results that the UVR8^C27^ domain displays characteristic IDP-like properties. This also explains why UVR8^C27^ undergoes temperature-induced (i.e. at increased free energy) secondary structure formation. The previously identified VP-motif in the UVR8^C27^ peptide^19^ has also been identified to be a key binding site for COP1 and RUPs^17^. The VP-motif forms a PPII structure to around 20% abundance in the low free energy windows, indicating a relatively conserved structural element. The PPII structure frequently exist in IDPs and has been found to be favourable for protein-protein and protein-nucleic acid interactions and thus plays a major role in signal transduction and protein complex assembly^27,28^. The proline amino acid residue has been suggested to play a major role in the formation of PPII structures in proteins and appears to rigidify IDPs^31^. It is thus highly likely that amino acid residue P411 in the VP-motif stabilizes the PPII structure of the UVR8^C27^ peptide, thus maintaining the structural integrity of this domain. However, we are also aware that, in addition to attaining a PPII structure in the VP motif, it is difficult to separate such a secondary structure from the fact that the proline may provide extensive contacts with the C27 binding site on COP1 that would be difficult for other amino acids to replicate.

The significance of the P411 residue was further characterized experimentally by far-UV CD using the mutant peptide UVR8^C27P411A^. Experimentally, the UVR8^C27P411A^ mutant peptide showed the same behaviour and properties as the wild type UVR8^C27^ peptide when H_2_O was used as the solvent. However, in presences of TFE, the UVR8^C27P411A^ peptide exhibited a significant structural change, in contrast to the wild type UVR8^C27^ peptide. The large increase in α-helix content found for the UVR8^C27P411A^ peptide in TFE implies that P411 is not only a key residue in COP1 binding but again plays a critical role in preserving the non-structured form of the UVR8^C27^ domain.

Interestingly, since both the low energy and the high energy states are represented by ensembles of many conformations, the single isodichroic point noted in CD experiments and supported by metadynamics simulations, indicate that UVR8^C27^ may readily switch between the more structured high energy conformations via the disordered global energy minima. These conformations are likely important for establishing the initial protein interaction between UVR8 and its interaction partners COP1 and RUP1/2. The ability of UVR8^C27^ to change structure would account for its ability to switch the interaction of UVR8 between COP1 and RUP. Hence, both experiments and metadynamics simulations suggest that the UVR8^C27^ domain is capable of interacting selectively with many other proteins and not only COP1 and the RUPs. Indeed, some recent studies have revealed new UVR8 interaction partners involving its C-terminus, namely WRKY36^35^ and BES1 and BIM1^36^. In fact, we expect there are several additional interaction partners that are yet to be discovered. Among these there are probably DNA-binding proteins that, similarly to WRKY36, may modulate the UV response on transcription at the promoter level^35,37^. This also suggests that UVR8-dependent UV regulation of development and morphology in plants is not solely mediated via COP1.

It has been suggested that the UVR8 C-terminal region is hidden from potential interaction partners when the UV-B photoreceptor is in its dimer form^13^. However, upon monomerization, the C-terminal would become exposed to the surrounding medium and thus be available for downstream interactions. This implies hidden and exposed states of the C-terminal region depending on the different molecular environments. Previous data on the relationship between dimer dissociation and exposure of the C-terminus of UVR8 is somewhat equivocal and two hypotheses have been proposed: first, the UVR8 C-terminus may be located distal to the interaction surface of the UVR8 dimer, as suggested by small angle X-ray scattering data^10^; secondly, a β-sheet structure of the C-terminus may be formed by interaction with the UVR8 N-terminus as indicated by analysing the UVR8 sequence using Weblogo^38^. However, our present study shows that the UVR8 C-terminal domain is highly tuneable to its surrounding environment. Hence, it is very likely that the C-terminus exists in different conformations in the hidden and the exposed states, and when binding to the different interaction partners (COP1 or RUPs).

We thus suggest that upon absorption of UV radiation, the UVR8 homodimer dissociates and the C-termini that are exposed to solution are in their non-structured unrestricted states, but in thermal equilibrium with more ordered high energy conformations that facilitate binding to the COP1 WD40 domain. Through the UVR8 interaction with COP1, the transcription factor HY5 is protected from COP1-dependent degradation and is thus able to induce downstream expression of UVR8-responsive genes (including *RUP1/2* gene expression^22^). Down-regulation of the UV response is thereby induced by the newly synthesized RUPs as they interact with the UVR8 monomer C-termini, most likely at higher affinity than that of COP1, subsequently “tuning” the UVR8 C-termini to a specific structure that promotes the reassembly of the UVR8 homodimer complex.

## Conclusions

In this work, the UVR8 C-terminal amino acid residues 397–423 (UVR8^C27^) was studied by CD and NMR spectroscopy together with bias-exchange meta-dynamics simulations. A disordered structure of UVR8^C27^ was verified from both experimental and theoretical results. From the theoretical studies, an inverted free energy landscape was revealed with a disordered UVR8^C27^ structure in the global energy minimum and more structured states at higher free energies, which were consistent with the experimental results. This established UVR8^C27^ as an intrinsically disordered protein. A high percentage of semi-disordered polyproline II (PPII) structure was found in the relatively low energy states *in silico*. A single point mutation P411A in the UVR8^C27^ peptide showed that the wild type P411 stabilized the disordered structure of UVR8^C27^ peptide that previously have been shown to be essential for binding to the COP1 and RUP1/2 proteins. The fact that UVR8^C27^ is an IDP makes it an ideal switch for accomplishing sequential binding of the UVR8 monomer with COP1 and RUP1/2 and the final reformation of the UVR8 dimer.

## Materials and Methods

### Simulation details

Molecular dynamics simulations of UVR8^C27^ were performed using the Amber99sb-ILDN force field^39^ with the TIP3P water model^40^. All simulations were performed using GROMACS 5.0^41^ together with PLUMED 2^42^. A time step of 2 fs was used together with LINCS constraints^43^. Van der Waals and electrostatic interactions were implemented with a cut-off at 1.0 nm, and long-range electrostatic effects were treated with the particle mesh Ewald method^44^. All simulations were carried out in the canonical ensemble at constant volume and by thermosetting the system with the Nosé-Hoover thermostat^45,46^ using a relaxation time of 1 ps. The atomic coordinates and the energy were saved every 2 ps. In terms of the metadynamics setup^47^, one-dimensional Gaussian functions of height 0.2 kJ/mol were added along the respective collective variables every 4 ps, and exchange of the bias potentials were attempted every 20 ps. Adding the Gaussian functions allows for the successive exploration of energetically difficult-to-access regions for a certain structural feature, one at the time. The starting conformations were built from MOE 2015.10^48^ followed by energy minimization. The UVR8^C27^ peptide was fully protonated in order to mimic the conditions at pH 6 and solvated with 3933 water molecules and 8 NaCl in a cubic box of 125.0 nm^3^ volume. The simulations were run for 500 ns on seven replicas (giving a cumulative simulation time of 3.5 µs) at 330 K. Convergence was reached after 30 ns in each replica. The (collective variable (CV)) space was divided in a grid cluster, and the free energy of each was estimated by a weighted histogram analysis method (WHAM) approach^49^.

### Collective variables

Bias-exchange metadynamics (BEM) consists of running several replicas of the same molecular dynamics simulation in parallel, each replica being biased by a different history–dependent potential acting on only one of the relevant CVs that describe the system^50^. In the simulations, we periodically allow for exchange between the bias potentials. Thereby we are able to fully sample the importance and relevance of different structural variables (described below) on the complex free energy landscape. The calculations were performed using seven replicas with 7 CVs.

*AlphaRMSD*, *ParaBetaRMSD*, *AntiBetaRMSD*

Three of the CVs (CV_1_: AlphaRMSD, CV_2_: ParaBetaRMSD and CV_3_: AntiBetaRMSD) are defined by the equations:

$${\rm{S}}=\sum _{a}c[RMSD({\{{R}_{i}\}}_{i\in {{\rm{\Omega }}}_{a}},\{{R}_{0}\})]$$

$$n(RMSD)=\frac{1-{(RMSD/0.08)}^{8}}{1-{(RMSD/0.08)}^{12}}$$

where *c* is a function switching smoothly between 0 and 1, the *RMSD* measures the root mean-square deviations in nm and ${\{{R}_{i}\}}_{i\in {{\rm{\Omega }}}_{a}}$ are the atomic coordinates of the backbone N, Cα, C, O and Cβ atom positions in a set of ${{\rm{\Omega }}}_{a}$ of six residues of the protein, while $\{{R}_{0}\}$ are the corresponding atomic positions of an ideal α-helix or β-sheet conformations. The CVs hence count the number of fragments of 6 residues belonging to α-helix or β-sheet structures, by computing their RMSD with respect to the ideal conformation structures (the average α-helix, parallel, and antiparallel β-sheet from the STRIDE database). In Gly residues, the Cβ is missing and the corresponding hydrogen is used instead.

### Contact Number

CV_4_ and CV_5_ are used to quantify the number of contacts between the side chain heavy atoms of different residues, and are defined as:

$${C}_{N}=\sum _{i,j}{C}_{ij}$$

with

$${C}_{ij}=\frac{1-{(\frac{{r}_{ij}}{{r}_{0}})}^{n}}{1-{(\frac{{r}_{ij}}{{r}_{0}})}^{m}}$$

where ${{\rm{r}}}_{{\rm{ij}}}$ is the distance between atoms or groups ${\rm{i}}$ and ${\rm{j}}$, r_0_ is the distance defining two atoms to be in contact and m and n are exponents that allow for tuning the smoothness of the function. CV_4_ takes into account hydrophobic contacts.${r}_{{\rm{ij}}}$ are the distances between the side chain heavy atoms i and j of residues V401, P403, A404, A408, V409, V410, and P411 in the UVR8^C27^ peptide. The value of r_0_ is 4 Å, which is an estimate of the average contact distance between side chain heavy atoms in the hydrophobic core of a protein. CV_5_ counts salt-bridge contacts, where ${r}_{{\rm{ij}}}$ are the distances between negative residues, and arginines and lysines. The value of r_0_ is 4.5 and 3.5 Å for CV_4_ and CV_5_, respectively.

### AlphaBeta similarity

CV_6_ and CV_7_ correspond to the χ_1_ and χ_2_ side chain dihedral angles, respectively, for hydrophobic and polar amino acids. These CVs are designed to enhance the side chain packing, which is crucial for protein folding. The CVs are defined as:

$$A{B}_{sim}=\sum _{i}\frac{1}{2}[1+\,\cos ({\chi }_{i}-{{\chi }_{0}}^{ref})]$$

where ${{\chi }_{0}}^{ref}$ is the mean value of the corresponding dihedral angle from a library of folded proteins extracted from the PDB.

For the UVR8^C27^ peptide, the Gaussian widths used in these bias-exchange simulations are 0.2, 0.2, 0.2, 2.0, 0.65, 0.5, and 0.2 for CV_1_–CV_7_, respectively.

### NMR spectroscopy

Natural abundant ^1^H-^15^N HSQC spectrum was recorded using a Varian INOVA 800 MHz NMR spectrometer at 25 °C for a synthetic UVR8^C27^ peptide encompassing amino acids GKSW VSPAE RYAVV PDETG LTDGS SKG (UVR8 aa 397–423). The NMR sample was prepared in deionized water and 10% D_2_O with a peptide concentration of 2 mM at pH 6. The data was acquired using 1024 complex points in the ^1^H dimension and 128 complex points in the ^15^N dimension. To gain high intensity H-N signals, the number of transients was set to 64. Processing was carried out in nmrPipe^51^ using the following scheme: First the free induction decay (FID) was subjected to a H_2_O solvent filter followed by a cosine-bell weighting function after which zero-filling was applied. After the mentioned procedure the FID was transformed to frequency domain by fast Fourier Transform. First-order polynomial baseline correction was performed to retrieve the final spectrum. Visualization was done in Sparky 3^52^. Overlapping resonances in the ^1^H-^15^N HSQC spectrum at 25 °C could be resolved at 8 °C confirming no peak splitting. Furthermore, 2D TOCSY spectra at 25 °C and 8 °C in combination with integration of selected NH signals in ^1^H-^15^N HSQC spectra support the above-mentioned statement.

### Circular dichroism

All measurements were conducted with a 33 μM peptide sample in deionized water (pH 6) using a Chirascan CD spectrometer (Applied Photophysics) equipped with a Quantum Northwest Peltier unit. Spectra were acquired using a bandwidth of 1 or 0.8 nm between 260–185 nm averaging 0.8 s per point. Each spectrum is the average of five repeats. The 1 mm cuvette was sealed using parafilm to avoid evaporation during thermal scan. Spectral reconstruction was performed using the CDSSTR algorithm and basis set number 6, and computed using Dichroweb^53,54^. All data were plotted in Origin.

### Material

The peptides UVR8^C27^ (aa 397–423; GKSWVSPAERYAVVPDETGLTDGSSKG) and UVR8^C27^ P411A (UVR8^C27P411A^; GKSWVSPAERYAVVADETGLTDGSSKG), were synthesized and purchased from GenScript at a purity >95%. Trifluoroethanol (99.5%) was purchased from Sigma –Aldrich.

## Supplementary information

Supplementary Information
