# Supplementary material for: Proline 411 biases the conformation of the intrinsically disordered plant UVR8 photoreceptor C27 domain altering the functional properties of the peptide
Source: Sci Rep. 2019 Jan 28;9:818. doi: 10.1038/s41598-018-37005-8 (PMC6349876; doi:10.1038/s41598-018-37005-8)
Supplement: Supplementary file 1 — Supplementary Information [file 41598_2018_37005_MOESM1_ESM.pdf]

Supplementary Information for:

Proline 411 biases the conformation of the intrinsically disordered plant UVR8 photoreceptor C27 domain altering the functional properties of the peptide

Min Wu, Daniel Farkas, Leif A. Eriksson, Åke Strid

Corresponding author: Åke Strid
Email: ake.strid@oru.se.

Supplementary Information

Fig. S1. Convergence of the energy surfaces as a function of different collective variables for $UVR8^{C27}$.
Fig. S2. Correlation between free energies estimated at 50 vs 100ns for $UVR8^{C27}$.
Fig. S3. Three dimensional representation of the free energy landscape of the $UVR8^{C27}$ peptides as a function of three collective variables.
Fig. S4. CD spectra and secondary structure composition for $UVR8^{C27P411A}$ peptide.
Table S1. Distribution of secondary structure elements as determined by reconstructions of CD spectra for the $UVR8^{C27}$ and $UVR8^{C27P411A}$ peptides at 10°C and 98°C.
Table S2. Distribution of secondary structure elements at 20°C determined by reconstructions of CD spectra for the $UVR8^{C27}$ and $UVR8^{C27P411A}$ peptides in presence and absence of trifluoroethanol (TFE)

Convergence of the free energy landscape

In order to determine the conformational properties of the UVR8 C-terminus, bias-exchange metadynamics (BE-METAD) simulations were employed. After 50 ns of simulation, most of the free-energy minima of the UVR8^{C27} system in each replica was filled and the bias potential V_G in each replica fluctuated around an average profile. The convergence of the free energy in each replica was monitored using the METAGUI package (*1*) plug-in to VMD (*2*) as illustrated in **Fig. S1**. The average free energies corresponded to the time intervals $\left(t_{eq}, t_{eq+all/_2}\right)$ and $\left(t_{eq+all/_2}, t_{all}\right)$, for the replicas among all collected variables (CVs) which are the total α-helix content (AlphaRMSD; CV_1), the total anti-β sheet content (AntiBetaRMSD, CV_2), parallel-β sheet content (ParaBetaRMSD, CV_3), the total number of hydrophobic contacts (Contact Number, CV_4), similarity of χ_1 dihedral angles to a reference value (χ_1, CV_5), similarity of χ_2 dihedral angles to a reference value (χ_2, CV_6) and the total number of salt bridge contacts (Salt Bridge, CV_7), where t_{eq} is 30ns and t_{all} is 330 ns for the UVR8^{C27} peptides. Based on these calculations, the two average free energy profiles were found to be within 1.5 k_bT of each other, indicating that the simulations had converged.

For the UVR8^{C27} peptide, a space defined by the four collected variables CV_1, CV_2, CV_3 and CV_4 was found to accurately describe the free energy of the systems. Adding another variable did not significantly improve the structural consistency within a cluster, but significantly reduced the average occupancy, lowering the quality of the free energy estimators. The set of clusters was thus defined by partitioning the four-dimensional CV space in small hyper-rectangles of sizes 0.2, 0.4, 0.4, and 1.2 for the UVR8^{C27} peptide. The free energies were estimated using the weighted histogram analysis method (WHAM) approach (*3*) where 1131 clusters were identified with free energies lower than 25 kJ/mol for the UVR8^{C27} peptide. These are representative of all the configurations explored for the systems. In order to further verify that convergence was reached for the UVR8^{C27} systems, the correlation between the free energies estimated at different filling time, were investigated (**Fig. S2**). The free energies of the clusters obtained at filling time t_1=50 ns have high agreements with the free energies obtained at filling time t_2=100 ns for UVR8^{C27}, indicating that the simulations had converged.

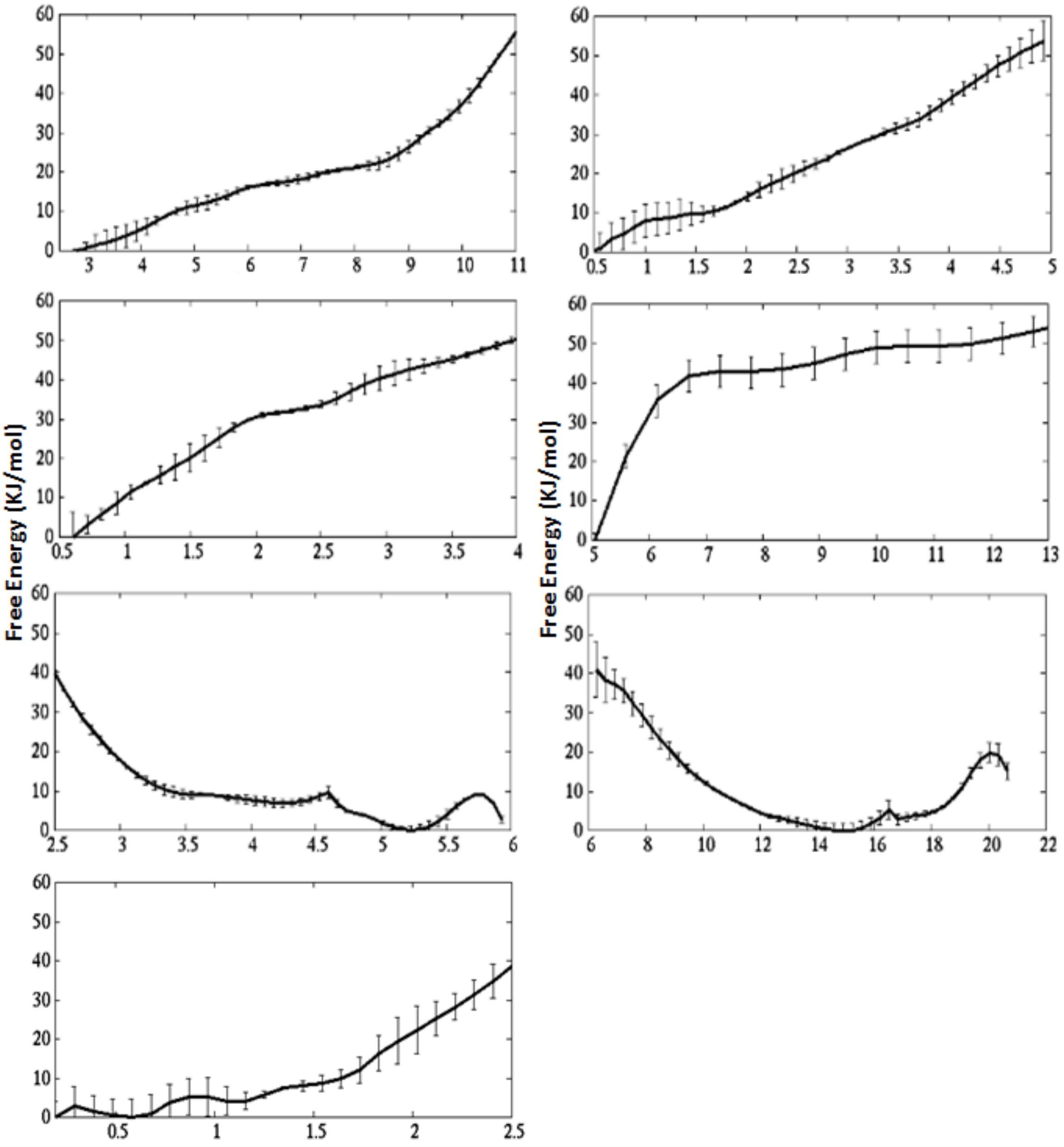

Fig. S1. Convergence of the energy surfaces as a function of the different collective variables CV1 – CV7 for UVR8[C27]. The error bars are within 1.5 $k_b T$ (approximately 4.11kJ/mol).

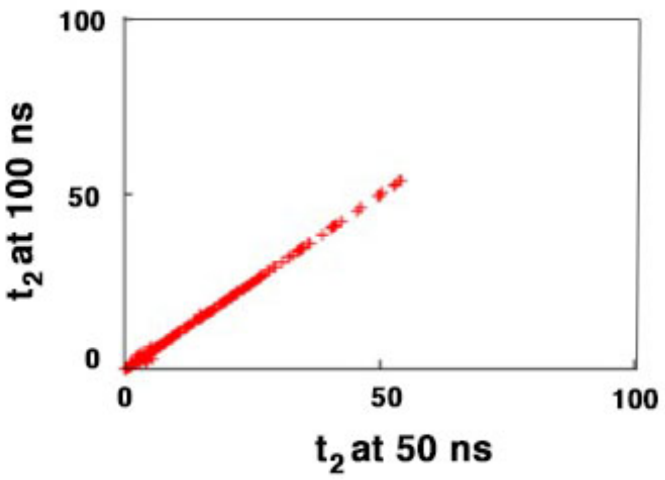

Fig. S2. Correlation between free energies estimated at 50 vs 100ns for UVR8^{C27}.

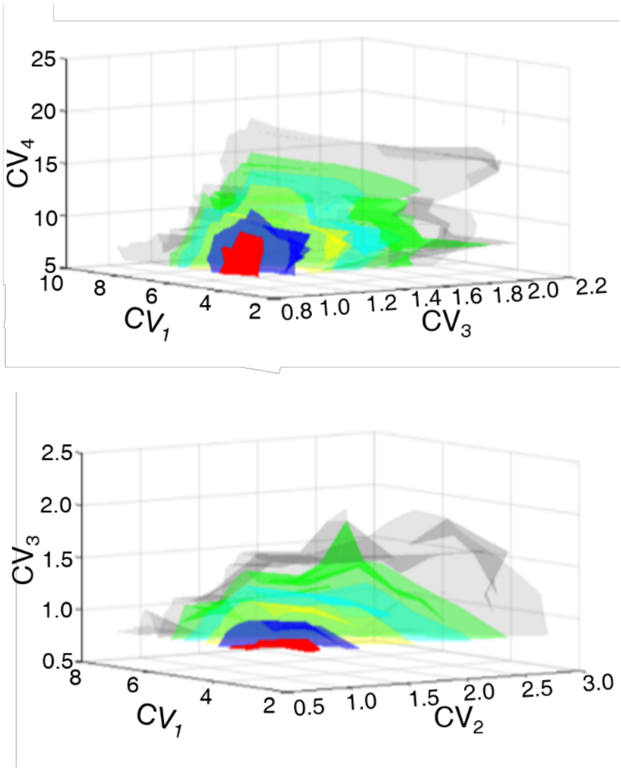

Fig. S3. Three-dimensional representation of the free energy landscape of the $UVR8^{C27}$ peptide as a function of three collective variables. Top panel: CV_1, CV_3 and CV_4, bottom panel: CV_1, CV_2 and C_3. See the Methodologies section of the main paper for definitions of the collective variables.

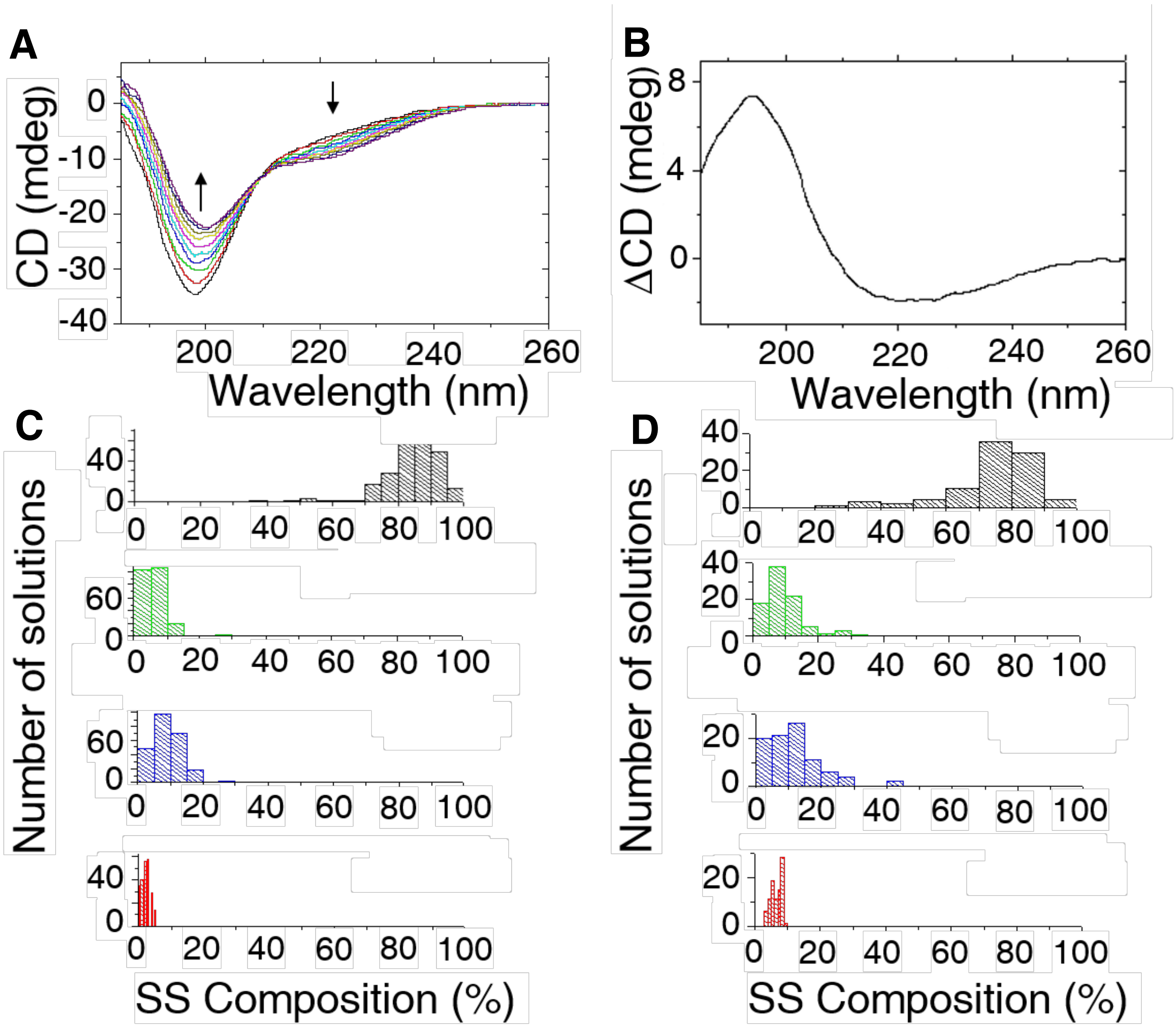

Fig. S4. CD spectra and secondary structure composition for the UVR8^{C27P411A} peptide. A) Far UV-CD spectra recorded at different temperatures for the UVR8^{C27P411A} peptide. The direction of spectral change with increasing temperature are indicated by arrows. The spectra were recorded for a 33μM solution in deionized water at pH6. B) Difference between far UV-CD spectra recorded at 98ºC and 10ºC, respectively, for the UVR8^{C27P411A} peptide. C) and D) Secondary structure (SS) compositions calculated from reconstructed CD spectra for UVR8^{C27P411A} at 10˚C (C) and 98˚C (D), respectively. Disordered structures are shown in grey, turns in green, β-strands in blue and α-helices in red.

Table S1. Distribution of secondary structure elements as determined by reconstructions of CD spectra for the UVR8^{C27} and UVR8^{C27P411A} peptides at 10°C and 98°C.

	Temp.(°C)	Helix (%)	Strands (%)	Turns (%)	Disordered (%)
UVR8^{C27}	10	3	9	6	82
	98	6	15	11	67
UVR8^{C27P411A}	10	2	9	5	84
	98	6	11	9	74

Table S2. Distribution of secondary structure elements at 20°C determined by reconstructions of CD spectra for the UVR8^{C27} and UVR8^{C27P411A} peptides in presence and absence of trifluoroethanol (TFE) as indicated.

	TFE (%) 20°C	Helix (%)	Strands (%)	Turns (%)	Disordered (%)
UVR8^{C27}	0	3	10	7	81
	25	8	13	8	72
	50	16	12	13	59
UVR8^{C27P411A}	0	2	10	6	81
	25	42	9	15	34
	50	60	9	15	17

References

1. Biarnes X, Pietrucci F, Marinelli F, Laio A (2012) METAGUI. A VMD interface for analyzing metadynamics and molecular dynamics simulations. *Comp Phys Comm* 183:203-211.
2. Humphrey W, Dalke A, Schulten K (1996) VMD: Visual molecular dynamics. *J Mol Graph Model* 14:33-38.
3. Roux B (1995) The calculation of the potential of mean force using computer simulations. *Comp Phys Comm* 91:275-282.
